# Scientometric analysis of trends in global research on acne treatment

**DOI:** 10.1097/JW9.0000000000000082

**Published:** 2023-07-28

**Authors:** Oleg Zolotarev, Aida Khakimova, Fakher Rahim, Engin Senel, Igor Zatsman, Dongxiao Gu

**Affiliations:** a Institute of Information Systems and Engineering Computer Technologies, Russian New University, Moscow, Russia; b Department of Anesthesia, Cihan University - Sulaimaniya, Kurdistan Region, Iraq; c Department of Dermatology and Venereology, Hitit University Faculty of Medicine, Corum, Turkey; d Research Department, Institute of Informatics Problems FRC CSC RAS, Moscow, Russia; e MIS School of Management, Hefei University of Technology, Hefei, Anhui, China

**Keywords:** Acne, acne treatment, acne vulgaris, publication trends, scientometrics, trend analysis

## Abstract

**Background:**

Acne or acne vulgaris is the most common chronic inflammatory disease of the sebaceous follicles.

**Objectives:**

The present study aims to identify the main lines of research in the field of acne treatment using reproducible scientometric methods. In this article, we reviewed the following research trends: facial acne, different antibiotics, retinoids, anti-inflammatory drugs, epidermal growth factor receptor inhibitors therapy, and associated diseases.

**Methods:**

The analysis of publications from the PubMed collection was carried out from 1871 to 2022. All data were analyzed using Microsoft Excel. The evolution of the terminological portrait of the disease is shown.

**Results:**

Trends in the use of various groups of antibiotics, retinoids, anti-inflammatory drugs, and photodynamic therapy for acne treatment have been found. There is a growing interest in clindamycin and doxycycline (polynomial and exponential growth, respectively). The effects of isotretinoin are also being studied more frequently (active linear growth). The publication of studies on spironolactone is increasing (linear growth). There is also a steady interest in the use of epidermal growth factor receptor inhibitors in the recent years. There is active research on acne and polycystic ovary syndrome (exponential growth).

**Limitations:**

Only articles in English were selected. The most frequent terms were considered.

**Conclusions:**

The dynamics of publication activity in the field of acne was considered. The aim of the current scientometric study was to analyze the global trends in acne treatments. The trend analysis made it possible to identify the most explored areas of research, as well as indicate those areas in dermatology in which interest is declining.

**What is known about this subject regarding women and their families?**

- Acne vulgaris negatively affects women’s life satisfaction.
- Acne vulgaris in women can manifest itself both in the adolescence and in the adulthood.
- Two-thirds of dermatologist visits for acne are women.

**What is new from this article as messages for women and their families?**

- Trends in the treatment of acne have been identified, including increases in publication on antibiotics and spironolactone.
- There is a notable loss in popularity of photodynamic acne therapy.
- The connection between acne and female diseases, including polycystic ovary syndrome and SAPHO syndrome, is being explored more frequently.

## Introduction

Acne is a chronic, inflammatory skin disease that causes spots and pimples, especially on the face, shoulders, back, neck, chest, and upper arms.^1^ According to the American Academy of Dermatology in 2013, more than 5.1 million individuals sought medical treatment for acne. This amount was comparable to the lost productivity from skin cancer and acne affects between 40 and 50 million Americans.^2^

Acne can be caused by various pathogenic factors such as follicular hyperkeratosis, increased sebum production, bacterial growth, and inflammation.^3^ People with acne often have low levels of depression, anxiety, low self-esteem, and generally a poorer quality of life, apart from its clinical severity.^4^ Various methods are used to treat acne, including photodynamic therapy, intense pulsed light, pulsed dye lasers, potassium titanyl phosphate lasers, infrared diode lasers, and broad spectrum continuous light sources (red light and blue-red light).^5^

Hormonal therapies such as spironolactone and combined oral contraceptives are important adjunctive therapies in women with acne. Spironolactone was created as an antihypertensive agent but has been used as an adjunctive acne therapy in dermatology since the 1980s. Spironolactone has an antiandrogenic effect.^6^ Spironolactone is a possible alternative to oral isotretinoin.^7^ Although dermatologists have recommended spironolactone for the treatment of acne in women for over 30 years, there are no reliable clinical trial data.^8^

Scientometrics is the knowledge of measuring research productivity.^9^ Scientometrics tries to describe science by using quantitative data related to the production of scientific literature^10^ and indexing databases such as Pubmed, ISI web of science, Scopus, etc.^11^

PubMed database is an electronic library of biomedical publications,^12^ which includes the Medline library^13^ and the Medical Subject Headings (MeSH) thesaurus.^14^ The PubMed is maintained by the National Center for Biotechnology Information^15^ and the National Library of Medicine,^16^ which in turn are part of the US National Institutes of Health.^17^ MEDLINE search strategies are based on subject headings or text words that are complemented by specific subject terms to provide an accurate search.^18^

Although acne is one of the most common diseases, there is no scientometric analysis published in the medical literature related to this disease. We conducted a scientometric assessment of acne and its treatment based on a trend analysis.^19^ Scientometric analysis allows to trace historical progress, assess trends in the development of medicine, and determine the most promising directions of its development.^20–22^

## Methods

Publications were identified by searching for the term “Acne Vulgaris” [MeSH] OR Acne [All Fields] in the search field using MeSH in PubMed. The search was carried out by titles and annotations of publications.

Statistics on word combinations with the term “acne” were taken into account.

Examples of a request to PubMed: ([akne] OR [acne]) AND ((“1861” [Date-Publication]: “1870” [Date-Publication])); (acne[Abstract]) AND isotretinoin [Abstract]. Requests to PubMed were completed on December 16, 2021 and February 12, 2022. Data analysis was carried out in December 2021, results were updated and expanded in December 2022.

The following metrics were chosen for the analysis: frequency of occurrence of terms by years in titles and abstracts, trend analysis (R-squared value, the square of the correlation coefficient).

We analyzed the terminology of articles for the period from 1999 to 2020. Due to the large number of articles, for analysis we selected keywords with a frequency of more than 4. In the resulting set, the terms with a frequency of less than 20 and those that occurred only in 1 year were rejected. The total number of terms was 67,285. In the resulting set of terms, thematic groups were identified and the frequency of occurrence of terms was analyzed.

The publication records were divided into 4 time periods: 1871 to 1950, 1951 to 1980, 1981 to 2010, and 2011 to 2020 (2022).

The analysis of the temporal dynamics was carried out, and the parameters of the trends were calculated. In this study, we considered only English-language papers.

To extract terms from a collection of publications, a python program was developed. The results were verified by experts taking into account the contexts that were also identified using the same program.

## Results

The evolution of the term AKNE (ACNE) was reviewed using PubMed over a 10-year period from 1871 to 2020. But for more informational content, separate periods were combined.

As part of the research into the evolution of acne terminology, 4 time periods have been identified: 1871 to 1950, 1951 to 1980, 1981 to 2010, and 2011 to 2020. Table 1 provides the cumulative incidence statistics for “acne” terms from 1871 to 2020.

**Table 1.** Evolution of the term acne

Term	1950	1980	2010	2020
Rosacea	10	15	20	21
Scar	1	5	10	13
Juvenile	3	7	8	
Chronic	1	2	3	
Fulminans		1	12	14
Severe		1	5	6
Persistent		1		2
Inflammatory			5	6
Pathogenesis			4	8
Adult			1	5
Pediatric			2	3
Propionibacterium			2	3
Inversa			3	
Vulgaris	46	165	276	299
Conventional				1
Genetics				1
Active				1
Mixed			1	
Propionibacterium	18			
Scrofulosorum	8			
Varioliformis	7			
Bacillus	2			

Analysis of the data shows that the term “acne vulgaris” is the most common diagnosis in identifying diseases associated with “acne.” At the same time, this term appeared in publications at the beginning of the last century and remains the most frequent diagnosis.

We divided the important terms into thematic groups and the frequency of occurrence of terms was analyzed. We chose the following thematic groups (% of terms in the total set of terms): “Acne treatment” (5.05%); “Acne disease” (17.79%); “Biomedical terms” (26.16%); “Scientific terms” (38.52%); and “Other words” (12.48%). The terms related to “acne,” constitute a fairly large part of all terms (22.84%). General scientific vocabulary prevails, and biomedical vocabulary is widely represented.

For further research, we have chosen the term groups “Acne disease” and “Acne treatment.” We were also interested in terms from the third group, comorbidities and their pathogenesis.

From the first group of terms for the trend analysis, the following acne type terms were selected: “facial acne,” “acne conglobate,” “chloracne,” “atrophic acne,” and “acne pustulosis.” We divided the second group of terms into subgroups: “acne and antibiotics,” “acne and retinoids,” “acne and anti-inflammatory drugs,” and “acne and photodynamic therapy.” From the third group of terms, we chose the following: “acne and the epidermal growth factor receptor (EGFR) inhibitors (EGFRis),” and “acne and comorbidities.”

In total, we screened out 19,105 relevant articles, which was about 5,946 more than the publication numbers of acne vulgaris (13,159 relevant articles). Most of these articles were published in journals that belong to the dermatology (10,523). The undisputed leader in terms of publication activity is the USA (31.8% of publications); in second place is Germany (7.7%) and in third place is the UK (6.9%). Also included in the top-10 are as follows: France (6.5%), Italy (6.2%), China (5.3%), Turkey (4.8%), India (4.8%), Japan (4.7%), and Canada (3.8%).

Before 2010, the field of acne has not attracted considerable consideration of scientists. However, the number of published articles on acne has been growing in the past decade. The number of citation has largely improved over time, reaching the highest in 2014 to 2016. The almost complete absence of publications in 2020 is due to the fact that the attention of scientists was riveted on the study of coronavirus. For further consideration, the articles published before 1946 were excluded. The remaining collection included 7,808 articles.

## Trends in publication activity related to acne

For further analysis, we have selected the most common words from the following topics: types of acne; acne and antibiotics; acne and retinoids; acne and anti-inflammatory drugs; acne and photodynamic therapy; acne and EGFRis; acne and concomitant diseases.

### Types of acne disease

The collocation “facial acne” appeared in publications in 1948. Despite the fact that the term of “facial acne” is not an official diagnostic code, the frequency of its use is actively exponentially increasing (y = 1.3868e^0.0581x^).

**Fig. 1.**
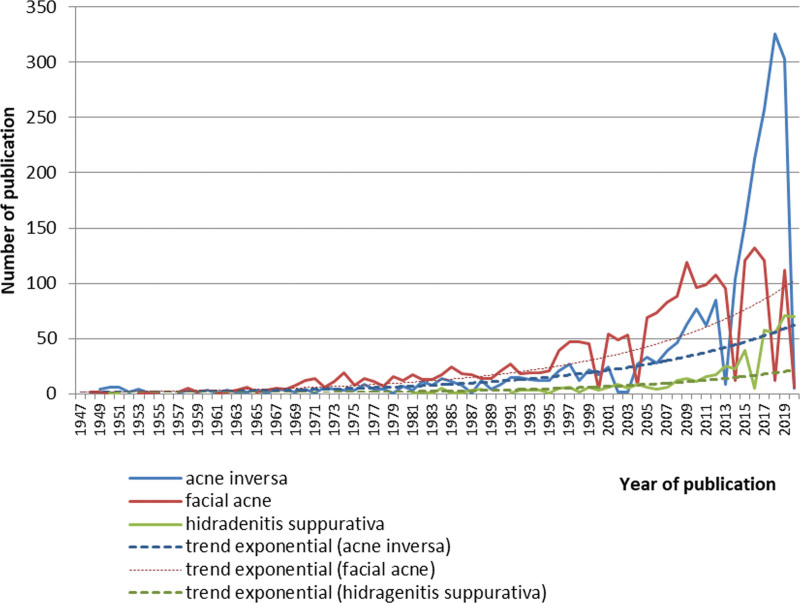
Trend analysis of publications by terms “facial acne.”

The considered term is characterized by a sharp exponential increase in the number of publications (Fig. 1). R^2^ is the square of the correlation coefficient. For “facial acne,” R^2^ = 0.734.

The same analysis was developed for collocations: “acne conglobate,” “chloracne,” “atrophic acne,” and “acne pustulosis.” The term “acne conglobate” appears in publications in 1931, “chloracne” in 1944, “atrophic acne” in 1960, and “acne pustulosis” in 1984. The terms “acne conglobate,” “atrophic acne,” and “acne pustulosis” differ by an increase in the number of publications. The number of publications with the terms “atrophic acne” and “acne pustulosis” is growing rapidly. The term “chloracne” indicates a powerful growth.

### Antibiotics for acne treatment

To analyze separately, we isolated tetracycline antibiotics (tetracycline, minocycline, doxycycline, and lymecycline), macrolides, and lincosamides (clindamycin, lincomycin, and erythromycin).

**Fig. 2.**
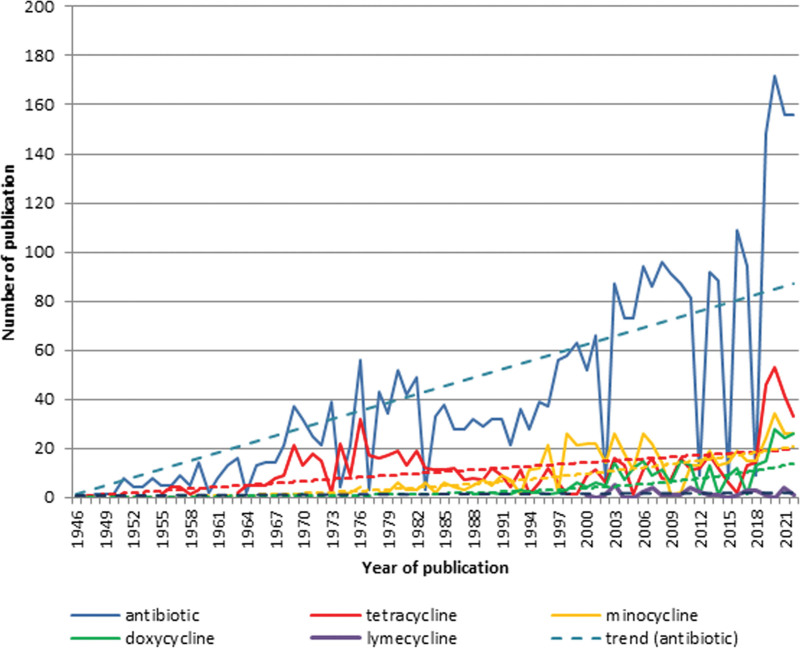
Trend analysis of publications by terms “antibiotic,” “tetracycline” (including commercial name “achromycin”), “minocycline,” “lymecycline,” and “doxycycline” (1946–2022).

In general, the increase in the number of articles on the term “antibiotic” with term “acne” since 1946 has been steadily linear, y = 1.1349x, R² = 0.784 (Fig. 2, upper line).

Minocycline is rather actively studied (since 1969, y = 0.001x^2.2813^, R² = 0.4815, weak power growth). Doxycycline shows some exponential growth since 1969 (y = 0.1417e^0.0595x^, R² = 0.6208). Studies of tetracycline (achromycin) still attract the attention of researchers (since 1955, y = −0.0001x^2^ + 0.2661x, R² = 0.1426, low polynomial growth). Research of lymecycline has been growing in recent decades (linear growth, y = 0.0119x + 0.8612, R² = 0.0086) (Fig. 2).

**Fig. 3.**
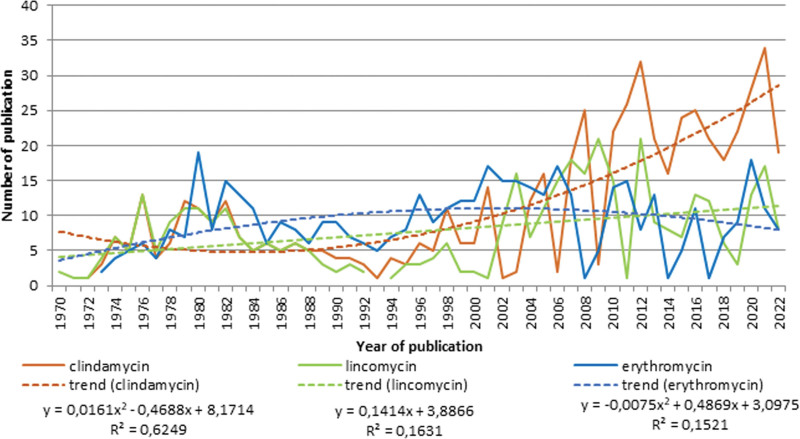
Trend analysis of publications by terms of “clindamycin,” “lincomycin,” and “erythromycin” (1970–2022).

In another group of antibiotics, interest in clindamycin is growing (R² = 0.6249, polynomial growth). The number of publications related to lincomycin in acne treatment (R² = 0.1631, linear growth) is slightly growing. The number of publications related to acne and erythromycin falls (R² = 0.1521, polynomial decay) (Fig. 3).

### Retinoids for acne treatment

**Fig. 4.**
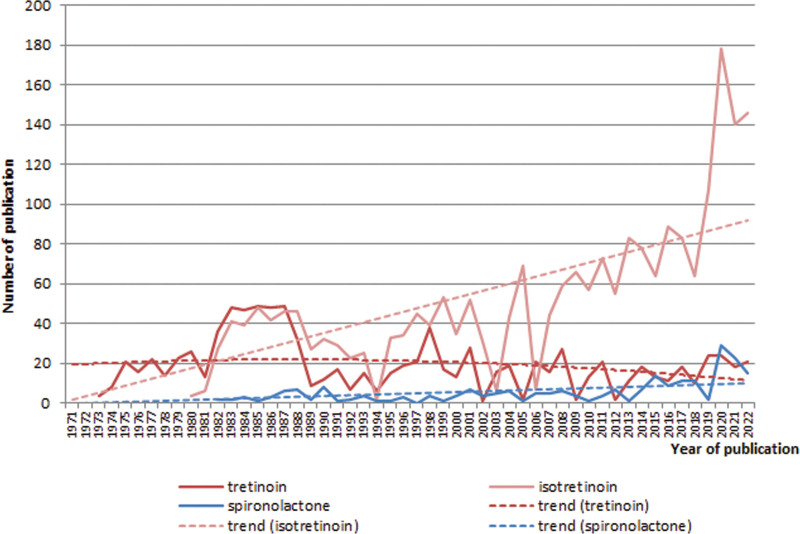
Trend analysis of publications by terms “tretinoin,” “isotretinoin,” and “spironolactone” (1970–2022).

It can be seen from Figure 4 that the studies of tretinoin (retinoic acid) are decreasing (polynomial decay, y = −0.0091x^2^ + 0.3242x + 19.086; R² = 0.0566), while isotretinoin’s study, by contrast, is actively growing (linear growth, y = 1.7864x; R² = 0.8441) (Fig. 4). The studies of spironolactone are also increasing (linear growth, y = 0.1899x, R² = 0.6356).

We considered also adapalene and tazarotene for acne treatment. As the trend analysis shows, adapalene research is actively growing (power trend, y = 1.278x^0.6913^; R² = 0.3072). Studies of tazaroten significantly reduced (polynomial decay, y = −0.0132x^2^ + 0.452x + 0.9479; R² = 0.0822).

### Anti-inflammatory drugs and photodynamic therapy for acne treatment

Benzoyl peroxide (BPO) exhibits strong polynomial growth since 1964 (y = 0.007x^2^ + 0.1894x + 1.0534; R² = 0.5252).

The literature data indicate a significant effect of azelaic acid on the pathogenesis of acne. It gives a pronounced anti-inflammatory effect, and exhibits antibacterial activity. Azelaic acid shows a weak power growth since 1983 (y = 0.0456x^1.1882^; R² = 0.2115).

**Fig. 5.**
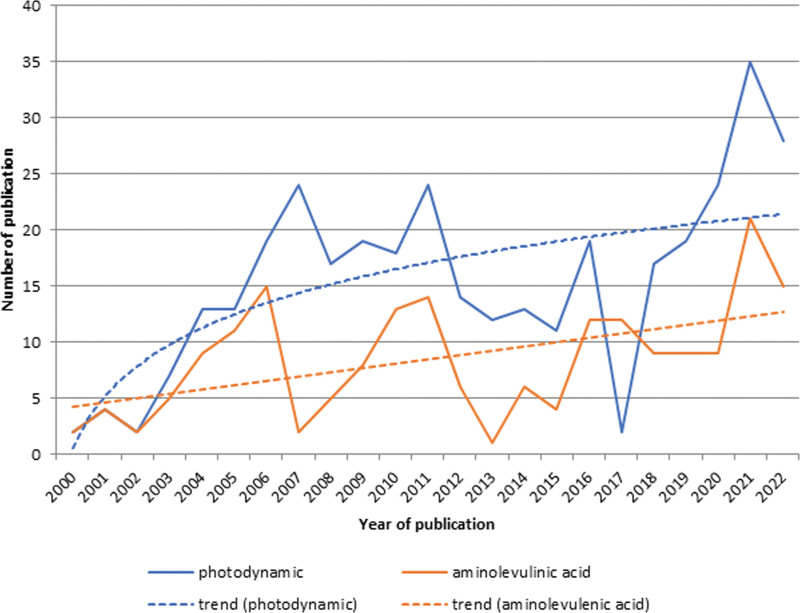
Trend analysis of publications by terms “photodynamic” and “aminolevulinic acid” (2000–2022).

Research in the field of photodynamic therapy of acne, actively conducted in the 2000s, is losing popularity in 2012 to 2017, then research resumed (y = 6.642ln(x) + 0.5752, R² = 0.4098, logarithmic growth) (Fig. 5). Aminolevulenic acid is also being investigated unregularly; however, it still remains in the research area (y = 0.3844x + 3.8221, R² = 0.2562, linear growth) (Fig. 5).

### Acne and epidermal growth factor receptor inhibitors

**Fig. 6.**
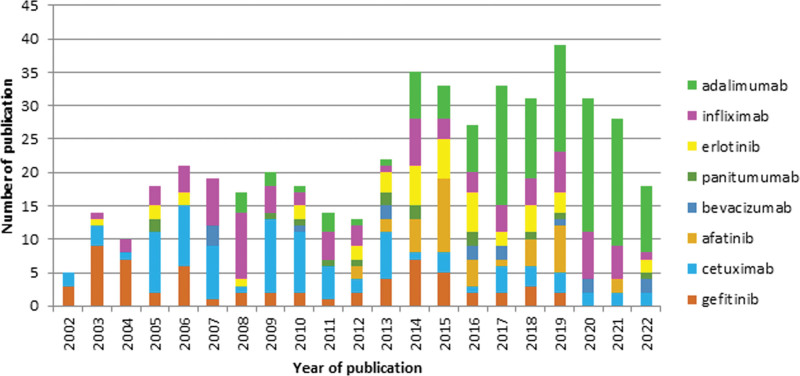
Trend analysis of publications by terms related to acne as a consequence of the application of EGFRis therapy (2002–2022). EGFRis, epidermal growth factor receptor inhibitors.

In recent years, a growing number of studies have been devoted to the connection between acne and EGFRis (Fig. 6). Overexpression of EGFR is found in some cancers. Inhibition of EGFR can impair tumor growth.^23^ However, EGFRis cause severe dermatological adverse events. Currently, there are 2 groups of drugs that block the EGFR: tyrosine kinase inhibitors (gefitinib, erlotinib, and afatinib) and monoclonal IgG 1 antibodies to the EGFR (cetuximab and panitumumab).^24^

### Acne and related diseases

**Fig. 7.**
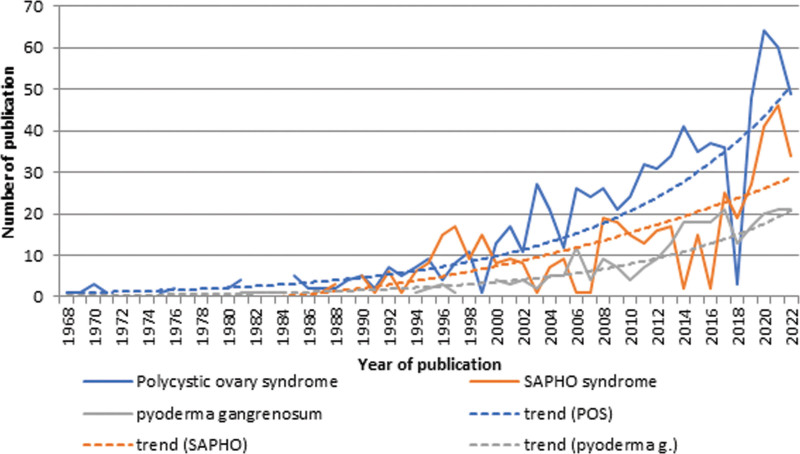
Trend analysis of publications by terms related to acne and associated diseases (1968–2022).

Research is actively conducted in the field of polycystic ovary syndrome (exponential growth, y = 0.8256e^0.0748x^, R² = 0.805). Slightly weaker, but synovitis acne pustulosis hyperostosis osteitis (SAPHO) syndrome (y = 0.0135x^2^ – 0.2234x, R² = 0.5152, polynomial growth) and pyoderma gangrenosum (y = 0.2467e^0,0806x^, R² = 0.8719, exponential growth) are also being actively studied (Fig. 7).

## Discussion

The present study explored the scientometric analysis of global research on acne treatment by analyzing the published literature from 1871 to 2020.

The terminological portrait of the disease is undergoing evolution. Even Aristotle and Hippocrates recognized this disease and described its symptoms. The modern name “acne” apparently comes from the Greek “Ακμή” in the works of Aetius (5th century BC). In the 9th century, Willan and Bateman identified the main variants of acne: simplex, punctata, indurata, and rosacea. In 1840, Fuchs introduced the terms acne vulgaris, acne mentagra, and acne rosacea, since then the name acne vulgaris has been used.^25^

The terms facial acne, acne inversa, atrophic acne, acne pustulosis are characterized by a sharp exponential increase in the number of publications.

Because the term “acne vulgaris” has a long history, there are various options for its use in practice. The general term “acne” describes a variety of manifestations of the disease, the nature of which is clarified by an additional definition. The second term “vulgaris” is rather convenient for practical use.

The antibiotics for systemic and external use have remained the basic drugs for many decades for most patients with acne. They inhibit the synthesis of bacterial proteins. These drugs are most effective in the inflammatory stage of acne. Although acne is not an infectious disease, oral antibiotics have remained a mainstay of treatment over the last 40 years. The anti-inflammatory properties of oral antibiotics, particularly the tetracyclines, are efficacious in treating inflammatory acne lesions.^26^

At various times, different antibiotics were used for acne treatment:

- From 1946 to 1955, in the 1950s: chloromycetin, aureomycin, and signemycin
- From the 1950s: streptomycin, oleandomycin, and novobiocin
- From the 1960s: oxacillin
- From the 1990s: azithromycin and nadifloxacin.

Antibiotics targeting Cutibacterium acnes (formerly Propionibacterium acnes) have been the mainstay in acne treatment for the past 4 decades. Among them, macrolides, clindamycin, and tetracyclines are most often prescribed. Antibiotic use is used to control the colonization of Cutibacterium acnes and the cascade of inflammation.^27^

Clindamycin is most actively studied. Minocycline, doxycycline, lincomycin, and lymecycline are being actively studied. Studies on tetracycline and erythromycin are being reduced.

Antibiotics have been used in the treatment of acne since 1960. Approximately 20 years later, specialists faced the problem of Propionibacterium acnes resistance to the effects of macrolides and tetracyclines, the highest resistance was found for erythromycin, and the lowest for lipophilic tetracyclines (doxycycline and minocycline). Erythromycin was previously used when it was impossible to use tetracyclines. Its use is usually limited to use in pregnant women, nursing women, and children. Derivatives of tetracycline (doxycycline and minocycline) are widely used. Topical use of clindamycin is relevant, especially in combination with BPO and tretinoin. To prevent resistance, the combination of clindamycin with BPO or tretinoin is preferred in the external treatment.^28^

Recent evidence-based guidelines for acne, including those from the American Academy of Dermatology and the European S3 guidelines from the European Dermatology Forum, have agreed that retinoids have an important role in the treatment of this widespread disease.^29^ The American Academy of Dermatology guidelines state that “retinoids are the core of topical therapy for acne because they are comedolytic, resolve the precursor microcomedone lesion, and are anti-inflammatory.”^30^

In the field of the use of retinoids, studies of isotretinoin and adapalene are actively growing. Studies of tretinoin (retinoic acid) and tazaroten fade.

Topical retinoids should be used in combination with BPO to optimize results in patients. Adapalene is a synthetic photo-resistant retinoid. Adapalene has excellent tolerance^31^ and is highly effective in reducing skin lesions.^32^ Tretinoin is more effective against acne than adapalene, but has a higher potential for skin irritation.^33^ Tretinoin has potent comedolytic, anti-inflammatory effects, but it also has the greatest potential for skin irritation of all agents. Tretinoin is inactivated by the concomitant use of BPO.

The high efficiency of oral isotretinoin in the treatment of acne is due to its effect on the main stages of the pathogenesis of the disease. The drug acts on retinoid receptors, which leads to the restoration of terminal differentiation of keratinocytes and proliferation of the epithelium of the sebaceous glands. Isotretinoin is also able to inhibit inflammatory mediators (leukotrienes), thus giving a pronounced anti-inflammatory and immunomodulatory effect.^34^

BPO has been well established as a common medication for acne vulgaris in many countries, where clinical data have been accumulated over a long time.^26^ BPO has a strong antibacterial effect, but causes significant dryness of the skin, contact irritant, and allergic dermatitis. The product is significant for long-term use, because bacteria are not resistant to it, which is ideal for combination therapy. It is one of the most preferred topical treatments.^35^ BPO has been an important component of topical therapy for acne vulgaris for over the 5 decades because of its ability to markedly reduce inflammatory acne lesions.^36^

Azelaic acid exhibits both antibacterial and comedolytic properties. Additionally, it is also a competitive inhibitor of the thioredoxin-reductase enzyme of Propionibacterium acnes, which affects the inhibition of bacterial DNA synthesis that occurs in the cytoplasm.^37^

Acneiform rash is the most common side effect of EGFRis. Tyrosine kinase inhibitors are currently applied in the treatment of nonsmall cell lung cancer with EGFR. Acneiform rash is the earliest and most characteristic side effect of EGFR inhibition.

The severity of an acne-like rash is caused by a specific inflammation of the sebaceous hair follicles.^38^ EGFRs are acne inducers and can cause changes in the treatment of cancer. In this regard, adequate and effective therapy for this side effect is of particular importance.

Different therapeutic strategies have been proposed for the effective management of dermatological adverse events associated with EGFRis therapy (cetuximab, erlotinib, lapatinib, gefitinib, panitumumab, etc).^39^ One of the reasons for the decline in interest in EGFRs may be the acneiform response they often induce.

Given the multifactorial pathogenesis of acne and the barriers to treatment, recent studies anticipate creating and trusting more treatment modalities in the future.^29^ Tan et al.^26^, in a review focused on the treatment options for patients with acne, showed that although the level of evidence on the safety of current therapies is low, new treatment modalities continue to be developed to treat patients with acne. In line with our findings, Bettoli et al.^30^ presented a 35 years of experience in acne treatment and showed a considerable increase in the treatment modalities over the study period. Given our finding, clindamycin was most actively studied, which also was reported in the recent studies.^39–41^ The results of this study were in line with other researches in the term of growing body of evidence on using minocycline against acne.^42–44^ Although recent studies reported that in the treatment of resistant moderate-to-severe acne,^45^ a retinoid may help, as showed in our study some controversies still exist.^46,47^

## Conclusion

In recent decades, an integrated approach to the study of acne and associated diseases that play an important role in the pathogenesis of acne has been widely practiced.

The immense growth in research publications relating to the acne disease treatment in the global context, and the significant trends pertaining to the multifactorial etiology and nature of the disease that makes it challenging to treat; however, these reasons make summarizing scientific research findings more essential now than ever before. In regard to, the aim of the current scientometric research was to collect and explain the probability and extent of the global trend of treatment modalities against acne. Precisely, being aware of the most recent trends of anti-acne treatment can help healthcare practitioners and policy makers recognize major factors in research output. Trend analysis allows to identify the most promising (rapidly developing) areas of research, and fix those areas in dermatology in which interest is declining.

## Conflicts of interest

None.

## Funding

Supported by RFBR and NSFC, project number 21-57-53018.

## Study approval

N/A

## Author contributions

OZ: Conceptualized the study, developed software. AK: Developed the methodology and drafted the article. FR: Revised the article. ES: Assisted with study design. IZ: Assisted with data analysis. DG: Assisted with critical revision of the article. All authors have read and approved the final article.
